# The Beat

**DOI:** 10.1289/ehp.119-a428b

**Published:** 2011-10-01

**Authors:** Erin E. Dooley

## Perc Clings to Dry-Cleaned Clothes

A new study finds that the solvent perchlorethylene, or “perc,” can accumulate in dry-cleaned clothes over repeated cleanings, building up the most in wool, cotton, and polyester; silk was largely unaffected.^1^ Occupational exposures to perc, used by 65–70% of the estimated 25,000 dry cleaners in the United States,^2^ have been linked to numerous adverse health effects. The study did not determine whether the levels found present a definite health risk, but the authors conclude they could lead to indoor air levels that exceed government recommendations.

**Figure:**
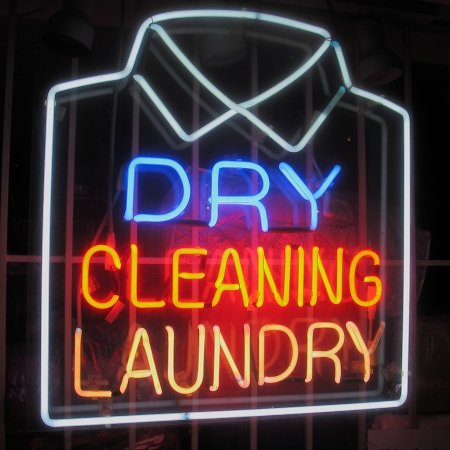
Perc is used by 65–70% of U.S. dry cleaners. Shutterstock.com

## Cadmium Settlement Reached

In September 2011, 26 retailers and suppliers reached a settlement with the California advocacy group Center for Environmental Health, agreeing to limit the use of cadmium in their jewelry products nationwide to no more than 0.03% by weight.^3^ The agreement goes into effect 31 December 2011. The companies also agreed to pay $1.03 million toward jewelry testing, future compliance testing, and other costs. Cadmium has been linked to cancer, kidney disease, lung disease, and bone weakness.^4^ It is not currently regulated in consumer products.

**Figure:**
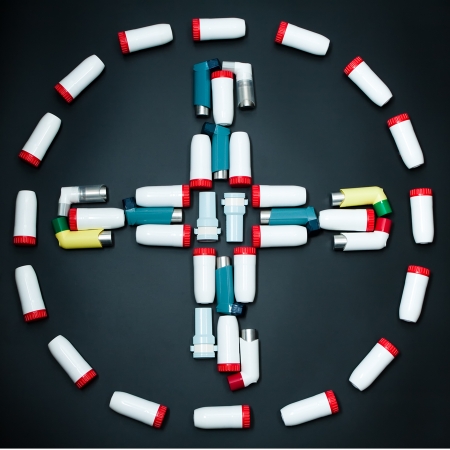
Elevated ground-level ozone is associated with asthma-related visits to emergency departments. Shutterstock.com

## Climate Change and Asthma

A new study estimates how future climate change may influence asthma-related emergency department (ED) visits on a local scale.^5^ Modeled changes in temperature and wind patterns predict an overall increase in levels of ground-level ozone, a respiratory irritant. The researchers used published climate models to predict summer ozone levels for five consecutive years in the 2020s, then compared them with levels and associated ED visits during the 1990s. They predict a 7.3% increase in ozone-related ED visits for asthma among children aged 0–17 in New York City.

## Possible Path toward Algaecides

Algal blooms produce toxins that can cause serious health effects and death in wildlife and humans and can contribute to oxygen-depleted marine “dead zones.” Using protein crystallography, researchers have now determined the structure of DapL, an enzyme in the l-lysine pathway that is essential to algal growth and development, potentially leading to the development of novel algaecides.^6^ The researchers suggest the enzyme and others in the l-lysine pathway could also be targeted to develop new antibiotics and herbicides.

**Figure:**
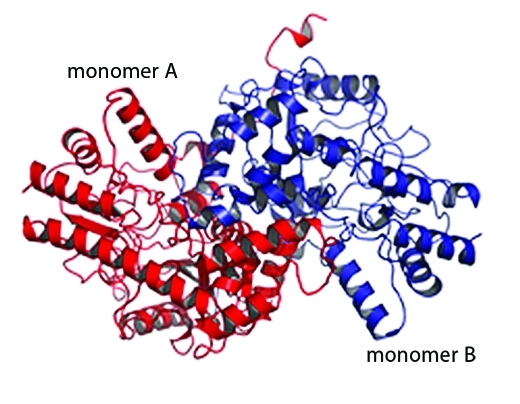
The DapL enzyme may be one key to short-circuiting algal blooms. Andre O. Hudson and Renwick C.J. Dobson

## Bolivia’s “Day of the Pedestrian”

On 4 September 2011 motor vehicle traffic came to a stop across Bolivia for the Day of the Pedestrian. News reports estimate at least 2 million cars were taken off the streets in nine cities, and public transit came to a halt.^7^ Air pollution is a serious problem in Bolivian cities more than 2,000 m above sea level: some locations have PM_10_ levels more than twice the average for Latin America and the Caribbean.^8^
